# Fire Performance of Cotton Fabrics Coated with 10-(2,5-Dihydroxyphenyl)-9,10-dihydro-9-xa-10-phosphaphenanthrene-10-oxide (DOPO-HQ) Zr-Based Metal-Organic Frameworks

**DOI:** 10.3390/polym15224379

**Published:** 2023-11-10

**Authors:** Qiuyue Wu, Manuel José Lis, Juan P. Hinestroza

**Affiliations:** 1 Institute of Textile Research and Industrial Cooperation of Terrassa (INTEXTER), Polytechnic University of Catalonia, Colón 15, 08222 Barcelona, Spain; qiuyueywu@gmail.com; 2 Department of Chemical Engineering, Polytechnic University of Catalonia, Colón 15, 08222 Barcelona, Spain; 3 Department of Fiber Science and Apparel Design, Cornell University, Ithaca, NY 14853, USA

**Keywords:** DOPO-HQ, Zr-based metal–organic frameworks, fire protection, cotton fabric

## Abstract

We investigated the performance of cotton fabrics coated with DOPO-HQ and Zr-based Metal–organic Frameworks when exposed to fire. The chemical structure of the cotton fabrics before and after the coating was characterized using FTIR spectroscopy, and the surface morphology of cotton and their combustion residues was probed via scanning electron microscopy. In our experiments, we used flammability tests and thermogravimetric methods to understand the burning behavior of the coated fibers, as well as their thermal stability. The cotton fabrics coated with DOPO-HQ and Zr MOFs exhibited shorter combustion times, had better thermal degradation properties, promoted the creation of heat-insulating layers, and exhibited improved smoke suppression behavior.

## 1. Introduction

Cotton, a natural hollow fiber with unique properties such as moisture absorption, air permeability, softness, comfort, and warmth retention, is one of the most used raw materials in the textile industry [1,2,3]. Since cotton fibers are inherently flammable and exhibit low thermal stability, significant efforts have been made to develop additives or coatings that can improve the properties of cotton when exposed to fire and high temperatures [4]. Several of these efforts have focused on developing halogen- and formaldehyde-free flame retardants [5,6,7]. Phosphorus-based flame retardants have proven to be effective coatings when exposed to fire, as they can generate phosphoric acid, metaphosphoric acid, and other derivatives that catalyze the dehydration and carbonization of cotton substrates covered with phosphoric acid derivatives. These coatings can also prevent the transport of oxygen and heat [8,9].

For the first time since 1972, 9,10-dihydro-9-oxa-10-phosphaphenanthrene-10-oxide (DOPO) reported by Sanko Chemical Co., Ltd., demonstrated enhanced anti-flammable properties among other available phosphorus-containing compounds [10]. A variation of DOPO, with formula 10-(2,5-dihydroxyphenyl)-9,10-dihydro-9-xa-10-phosphaphenanthrene-10-oxide, and known as DOPO-HQ, involves reacting DOPO and p-benzoquinone [11]. DOPO-HQ exhibits better chemical stability and heat resistance than DOPO due to its rigid aromatic structure and stable P-O-C bond. When DOPO-HQ decomposes at high temperatures, some of its derivatives, such as phosphoric acid contribute to suppressing the burning of cotton fibers [12,13].

Metal-organic frameworks (MOFs) [14] are hybrid compounds comprising inorganic metal nodes and organic ligands. Their controlled structure, unique porosity, and high specific surface area have offered possibilities for a myriad of applications in gas storage/separation [15], drug delivery [16], electrochemical sensing [17], catalysis [18], and flame retardant additives [19,20].

The interaction of MOFs and cellulose-based polymers offers many possibilities as most MOFs are also reticular structures implying that the crystalline structure can be maintained regardless of the molecular length of the ligand. This unique property allows unprecedented control of the porosity of MOF structures. Even better, the porosity and crystalline structure can be predicted with a greater degree of accuracy.

Cellulose comprises D-glucopyranose units linked by β-1,4-glycosidic bonds, resulting in three reactive hydroxyl groups per glucose unit. These hydroxyl groups can undergo several chemical modifications that can enable the grafting of active materials, such as metal–organic frameworks. For example, some of these chemical modifications include using a native moiety of the fiber that mimics MOFs’ complexing moieties. Another approach can exploit modifications to the surface chemistry of the fiber by adding an organic group mimicking MOFs’ complexing moieties. An additional approach includes integrating inorganic oxides into the surface of the fiber, so that they can play the role of inorganic components of a MOF structure.

MOFs have been incorporated as finishes into many textile matrices [21,22,23]. While undergoing thermal decomposition, the metal oxides generated from MOF particles on polymer substrate surfaces act as a physical barrier to protect the substrate from further burning and efficiently adsorb gases and smoke. Non-flammable gases formed by the thermal decomposition of MOFs can also dilute the concentration of flammable compounds [24]. Since their discovery by Lillerud [25], Zr-based MOFs have shown uniquely high thermal and chemical stability [26]. In particular, UiO-66 (UiO: University of Oslo) Zr-based MOF has been extensively studied and grown on the surface of cotton fibers [27]. UiO-66-COOH, which contains free pendant –COOH groups, can be synthesized via a hydrothermal procedure [28]. Water-based synthesis not only lowers manufacturing costs but also avoids using toxic solvents. UiO-66-COOH has good compatibility with cotton substrates [29]. The flame-retardant properties of cotton can also be improved after incorporating Zr-MOFs, as we previously reported [30].

To the best of our knowledge, there have not been public reports on using mixtures of DOPO-HQ and Zr-based MOFs to improve the flame-retardant properties of cotton. Therefore, in this article, we explore the structural characteristics and behavior shown by coated fabrics confronted with fire. We speculate that using sonication may help introduce DOPO-HQ molecules to the inside of the porous structure of Zr-MOFs. We also found that the resulting structure does not require additional reagents or co-adjuvants. We also explored coated fibers’ behavior as a function of the number of DOPO-HQ and Zr-based MOF layers deposited on the surface of cotton substrates.

## 2. Experimental Section

### 2.1. Reagent and Materials

Commercially available bleached 100% cotton twill fabrics with an areal density of 250 g/m^2^ were used. 10-(2,5-dihydroxyphenyl)-9,10-dihydro-9-xa-10-phosphaphenanthrene-10-oxide (DOPO-HQ) was purchased from Alfa Chemistry Co., Ltd. (Zhengzhou City, China). Zirconium chloride (ZrCl_4_) and trimeric acid (H_3_BTC) were purchased from Aldrich Chemical Co. (St. Louis, MI, USA). All reagents were employed as received without additional purification.

### 2.2. Sample Preparation of DOPO-HQ@Zr-MOF Microcomposites

We completely dissolved 2.4 g (10 mmol) of ZrCl_4_ in 50 mL of deionized water. Afterward, 2.2 g (10 mmol) of H_3_BTC was dissolved in 50 mL of deionized water using an UltraTurrax homogenizer (IKA, Staufen Germany) operating at 16,000 rpm. The two dispersions were mixed and refluxed in a sealed reactor at 100 °C for 16 h. We added 6.5 g (20 mmol) of DOPO-HQ powder to 170 mL of an ethanol/water mixture (1:1), ultrasonicated the mixture for 15 min, and left it overnight under vigorous magnetic stirring at room temperature.

**Figure 1:**
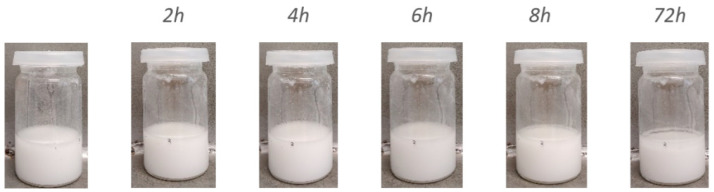
Digital photographs of DOPO-HQ@Zr-MOF dispersions as a function of time.

To study the gravitational stability of the DOPO-HQ@Zr-MOF dispersion, the sample was observed for an extended period of time, as shown in Figure 1. Digital photographs were used to visualize the evolution of dispersion over time. We observed that at ambient conditions, the DOPO-HQ@Zr-MOF suspension remained homogeneously dispersed for at least 8 h.

### 2.3. Functionalization of Cotton Substrates with DOPO-HQ@Zr-MOF

**Figure 2:**
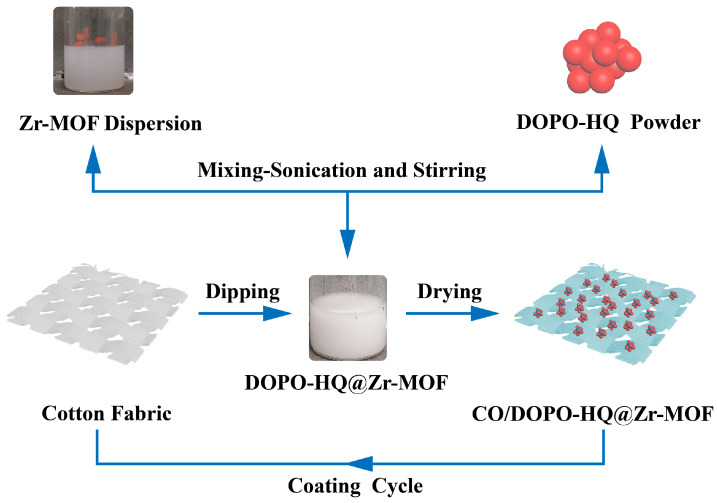
Graphical representation of one functionalization cycle for cotton fabrics with DOPO-HQ@Zr-MOF.

Cotton fabrics were exposed to the DOPO-HQ@Zr-MOF dispersion via dipping for 5 min. After dipping, the coated fabrics were dried in an oven for 4 min at 75 °C. This combination of dipping and drying was considered one dip–dry cycle, as illustrated in Figure 2.

Cotton fabrics were labeled as CO/DOPO-HQ@Zr-MOF-1 and CO/DOPO-HQ@Zr-MOF-10 depending on the number of dipping–drying cycles used (1 or 10). Each sample was weighed before and after applying the coating.

The add-on values (wt%) were calculated using Equation (1), and the results are summarized in Table 1. After depositing 1 layer and 10 multilayers of DOPO-HQ@Zr-MOF, the cotton fabrics attained weight gains of 2.82 and 12.03 wt%.

(1) $$Add-on\left(wt\%\right)=\frac{Last\;weight\;of\;specimen\left(g\right)-Initial\;weight\;of\;specimen\left(g\right)}{Initial\;weight\;of\;specimen\left(g\right)}*100$$

### 2.4. Characterization

UV-vis absorption measurements of DOPO-HQ, Zr-MOF, and DOPO-HQ@Zr-MOF specimens were performed using a UV-2401 PC spectrophotometer (Shimadzu, Kyoto, Japan). The UV experiments were performed at room temperature covering a wavelength range from 700 to 190 nm. The Fourier Transform Infrared (FTIR) spectra of the specimens were acquired with a Nicolet iS10 FTIR spectrometer (Thermo Fisher, Walham, MA, USA) in the 4000–500 cm^−1^ range with a spectral resolution of 4 cm^−1^.

Flammability tests were performed to investigate the burning behavior of the treated fabrics using a customized flame testing apparatus inspired by the norm ASTM D6413 standard. Textile samples, measuring 280 × 75 mm, were fixed and exposed to a vertical flame for 10 s. The burning process for each sample was video recorded.

To observe the surface morphology of the cotton fibers and the carbonized residues after the burning experiments, scanning electron microscopy (SEM), JSM-5610 (JEOL, Akishima, Japan) installed at the imaging platform center of the Polytechnic University of Catalonia (UPC) was used. Prior to SEM imaging, the specimens were coated with 10 nm of Au.

The thermal properties of specimens were evaluated using the Thermal Analysis System TGA 2 STARe (Mettler-Toledo, Columbus, OH, USA). All samples were loaded into an alumina holder and heated from 30 to 800 °C at a heating rate of 10 °C∙min^−1^ under an airflow of 50 mL∙min^−1^. Thermograms of weight, as a function of temperature, and the derivative of those thermograms (DTG) were used to obtain the thermal properties of coated and uncoated cotton fabric specimens.

## 3. Results and Discussion

### 3.1. UV-VIS Spectroscopy

**Figure 3:**
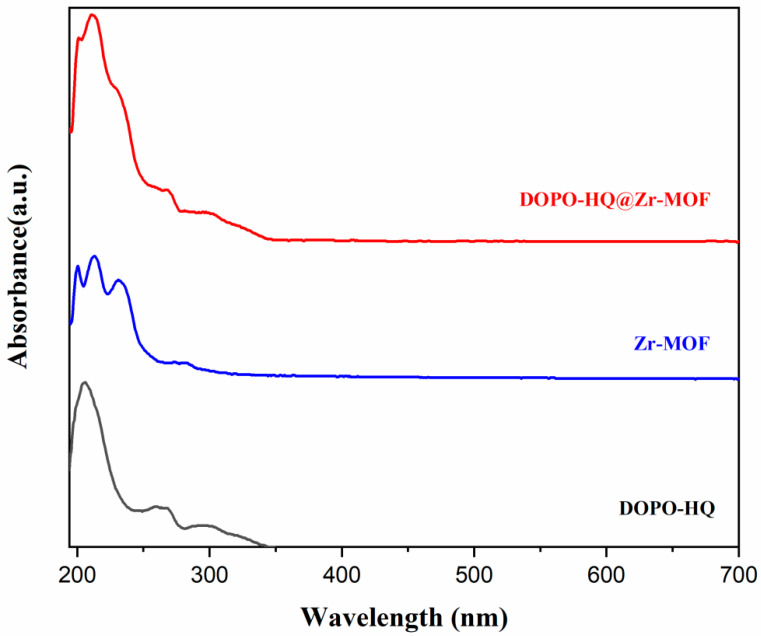
The UV-vis absorption spectra of DOPO-HQ, Zr-MOF and DOPO-HQ@Zr-MOF.

To confirm that an immiscible DOPO-HQ compound was mixed with the Zr-MOF structures, we used ultraviolet–visible absorption spectroscopy, as shown in Figure 3.

Strong absorption bands in the region below 260 nm confirm the π→π* excitation of the trimesic acid linker in the Zr-based MOF. These bands are in quantitative agreement with previous reports [31]. The maximum UV-visible absorption peak for Zr-MOF is found to be around 322 nm. The absorption peak for DOPO-HQ@Zr-MOF displayed a small red shift compared to previous reports [32]. The maximum absorption peak for Zr-MOF was located at 213.5 nm, yet it shifted to 211 nm in the spectrum of DOPO-HQ@Zr-MOF. The red shifting effect can be attributed to interactions between the MOF structure and DOPO-HQ that form a new complex structure. The adsorbed molecules are expected to modify radiation’s capability to be absorbed freely by the MOF structure, hence reducing the three possible excitation states to a combination of them.

### 3.2. FT-IR Characterization

**Figure 4:**
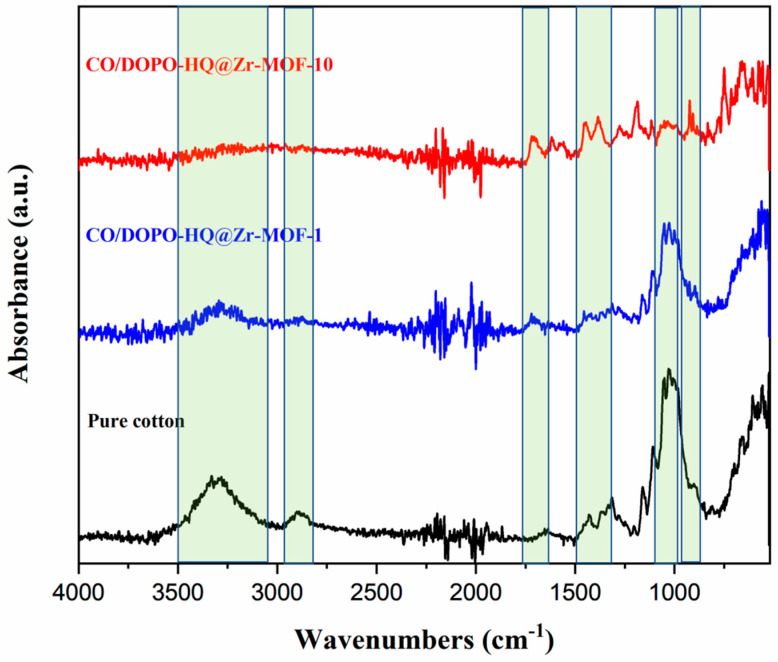
FTIR spectra of pure cotton (black), CO/DOPO-HQ@Zr-MOF-1 (blue) and CO/DOPO-HQ@Zr-MOF-10 (red). Bands of interest are highlighted in green.

Figure 4 shows the FTIR spectra for the specimens.

The FTIR absorbance spectra of the cotton specimens, before and after coating with DOPO-HQ@Zr-MOF, provide further confirmation of the chemical interaction between the coating and the cotton substrate. Peaks at 1025 cm^−1^, 1315 cm^−1^, 1363 cm^−1^, 1430 cm^−1^, and 2889 cm^−1^ in the untreated cotton specimen were associated with (CO) and (OH) stretching, C-O bending, C-H bending, CH_2_ bending, and C-H stretching vibrations [33]. The broad band around 3300 cm^−1^ was commonly assigned to the –OH groups and, as expected, it significantly weakened after applying the coating [34,35]. The peak observed at 1710 cm^−1^ was assigned to C=O stretching in the DOPO-HQ@Zr-MOF coating. The peaks at 748 cm^−1^, 1191 cm^−1^, and 1563 cm^−1^ were ascribed to P-C stretching, P=O vibration, and P-Ph stretching of DOPO-HQ [13,36,37]. The evolution of the peaks in the highlighted bands below 2000 cm^−1^ is a clear indication of the influence of the coating cycle number. This parameter can be used as a reliable metric if this process is eventually scaled up. All the spectra in Figure 4 are in quantitative agreement with previous literature reports [38].

### 3.3. Flammability Tests

**Figure 5:**
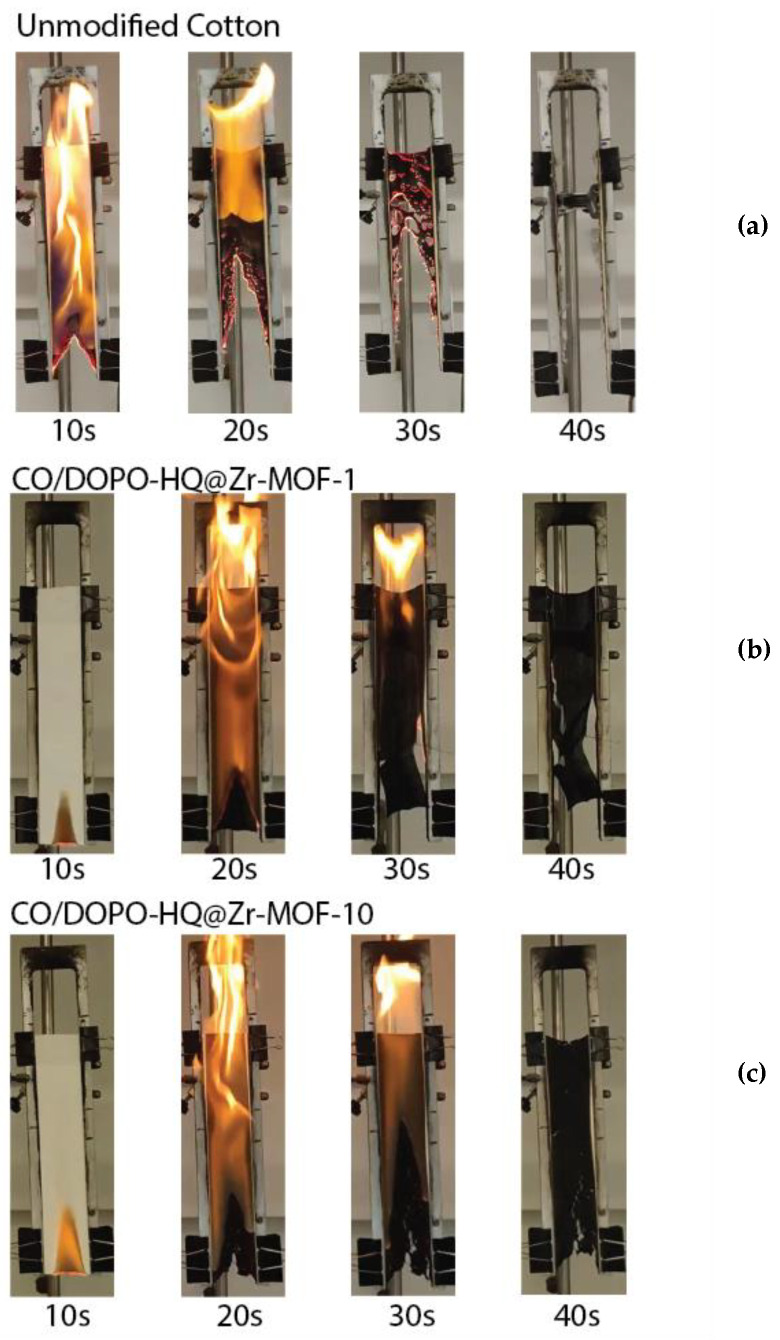
Digital screenshots of (**a**) unmodified cotton; (**b**) sample CO/DOPO-HQ@Zr-MOF-1; and (**c**) sample CO/DOPO-HQ@Zr-MOF-10 in the vertical flammability chamber as a function of time after ignition.

Coated and uncoated fabric specimens were exposed to a flame for 10 s before the ignition source was removed. The totality of the burning process was video recorded, and screenshots obtained from those videos are shown in Figure 5.

From the burning experiments, the untreated cotton sample violently burned and was consumed by the flame without generating residual char. The sample with only a one-layer deposition of DOPO-HQ@Zr-MOF was endowed with good char-forming properties. Consequently, the combustion time was shortened. As the cotton substrate was coated with additional layers of DOPO-HQ@Zr-MOF, there was a noticeable smoke suppression effect. Also, a denser and thicker carbon layer on the cotton surface was formed to protect the fibers’ structure. However, the differences between the CO/DOPO-HQ@Zr-MOF-1 and CO/DOPO-HQ@Zr-MOF-10 samples are not significant regarding the inhibition of flame spreading. The smoke suppression effect is related to the capability of MOFs to act as sorbents, which can be explained by the increase in layers. Nevertheless, the amount of DOPO-HQ seems insufficient to strongly modify FR behavior. The presence of char in the (b) and (c) samples can be attributed to the formation of inorganic oxides that increase char. This finding is in accordance with the proposal and results of several authors [24,25]. In Figure 5b,c, the retardant effect can be observed in the samples at 10 s. This effect can be attributed to differences in heat transfer characteristics created by the formed inorganic oxides [27].

### 3.4. Morphology Investigation

**Figure 6:**
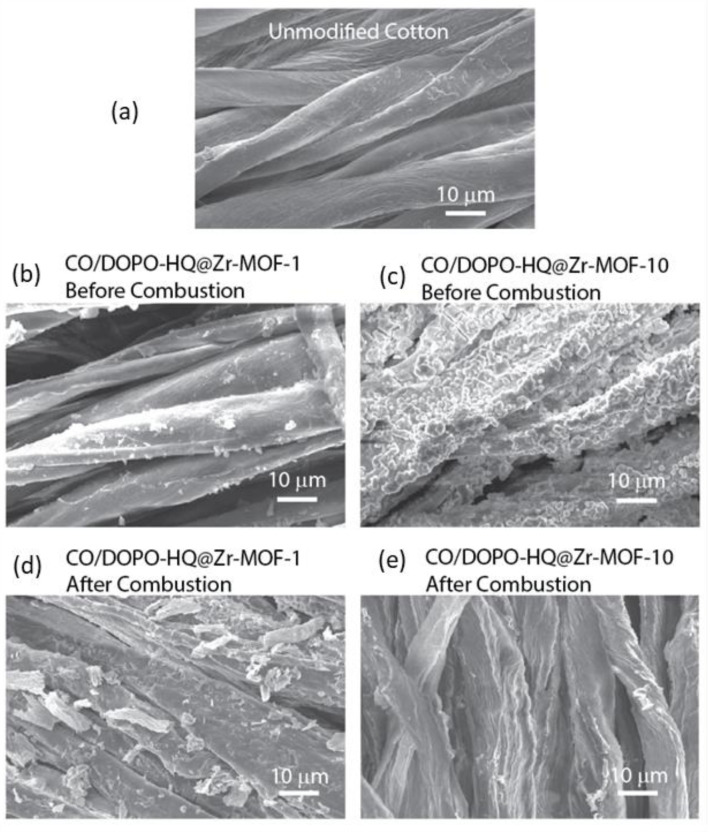
SEM images of (**a**) unmodified cotton; (**b**) sample CO/DOPO-HQ@Zr-MOF-1; and (**c**) sample CO/DOPO-HQ@Zr-MOF-10; (**d**) CO/DOPO-HQ@Zr-MOF-1 residual chars; and (**e**) CO/DOPO-HQ@Zr-MOF-10 residual chars.

Figure 6 shows SEM images of the surface morphology of cotton fabrics before and after combustion.

The surface of the untreated cotton fibers is initially flat and smooth, which allows for easy identification of DOPO-HQ@Zr-MOF clusters upon coating. Specimens coated with 10 cycles exhibit a very rough surface containing heat-insulating layers that protect the cotton substrate during combustion. These thick layers lead to non-flammable carbonized residuals upon combustion, basically consisting of Zirconium oxides, which can also be seen in SEM images [39]. It is worth noting that the fibers of the specimen CO/DOPO-HQ@Zr-MOF-10 appear to maintain some level of structural integrity after burning (Figure 6e), which agrees with previous reports.

### 3.5. Thermal Properties

**Figure 7:**
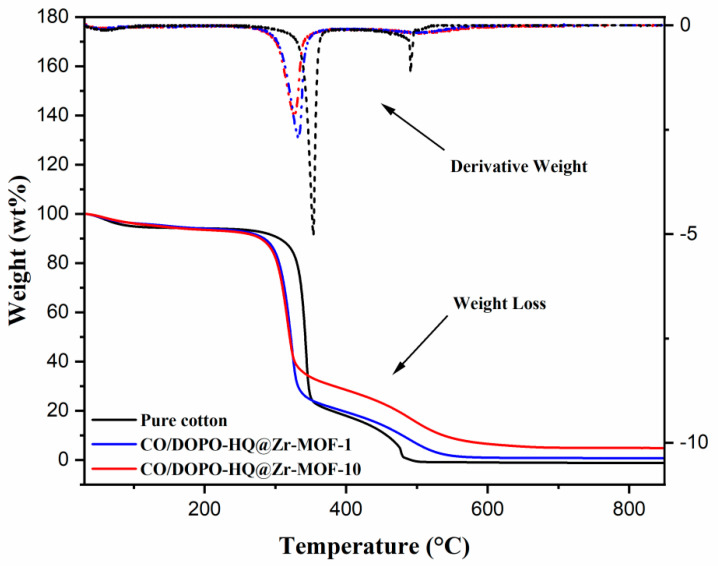
TGA and DTG curves of pure cotton (black), CO/DOPO-HQ@Zr-MOF-1 (blue), and CO/DOPO-HQ@Zr-MOF-10 (red).

In order to gain a better understanding of the thermal properties of the substrates, thermogravimetric analysis TGA was used to evaluate the weight loss as a function of temperature in a controlled atmosphere as shown in Figure 7 and Table 2.

Due to the hydrophilic nature of the cellulosic matrix, weight loss around 100 °C, can be attributed to moisture evaporation. The thermal decomposition process of each sample comprises two main stages. The first stage, the main thermal decomposition step, accounts for total weight loss. Pristine cotton shows a dehydration of the cellulosic polymeric chains, which facilitates the formation of char at 318–380 °C. The formed char is further oxidized to produce more gaseous combustible products at higher temperatures in the second stage.

According to the TGA experiments, the initial decomposition temperature (T_10%_) of the specimens coated with CO/DOPO-HQ@Zr-MOF-1 and CO/DOPO-HQ@Zr-MOF-10, was determined at 305.9 °C and 296.8 °C, respectively. Curiously, both values are lower than the decomposition temperature for pure cotton. This discrepancy can be attributed to the early decomposition of DOPO-HQ@Zr-MOF [40]. A quick glance at the thermograms indicates that the thermal degradation of cotton fabrics, under an air atmosphere, could be considered a two-stage process [41]. In the interval of 500–800 °C, the untreated cotton fabric was burned out without leaving any residuals. By contrast, the protective char formation of samples CO/DOPO-HQ@Zr-MOF-1 and CO/DOPO-HQ@Zr-MOF-10 yielded 0.77 wt% and 4.85 wt%, respectively. In sample CO/DOPO-HQ@Zr-MOF-10, the T_max_ increased from 479 to 484.5 °C and R_max_ decreased from 93.4 to 76.4 wt%/min compared to the untreated cotton specimen. These values indicate a noticeable improvement in the thermal behavior of the cotton samples once they were coated with DOPO-HQ@Zr-MOF. The stability at lower temperatures, as shown in the TGA results, supports the flame tests shown in Figure 5. During the first few seconds, samples with MOFs showed delayed burning behavior due to the quick formation of oxides. The presence of Zr in the polymeric structure on the textile substrate is known to catalyze carbonization, reduce heat release, and form C-C bonds during combustion [26,27,42], which helps convert the polymeric matrix into char (range of temperatures 600–800 °C, in Figure 7). This process has considerable stability (see Figure 5) and improves flame retardancy.

## 4. Conclusions

We used UV-vis spectroscopy to confirm that DOPO-HQ was adequately incorporated into porous Zr-MOF for preparing functional composite materials. Differences in chemical structures identified by FT–IR spectroscopy between pure cotton and the CO/DOPO-HQ@Zr-MOF-10 sample indicated that DOPO-HQ@Zr-MOF composites were effectively incorporated and interacted with cotton substrates. SEM images showed that compact multi-layers of DOPO-HQ@Zr-MOF composites were distributed uniformly on the surface of cotton fibers, allowing a carbonaceous insulation layer to form while retaining the original morphology of substrates during combustion. Flammability tests positively clarified that treated cotton fabrics with better smoke suppression properties were quite productive at shortening the combustion period. The results of TGA (DTG) demonstrated that by incorporating DOPO-HQ@Zr-MOF micro-composites, the thermal degradation of cotton fabrics was inhibited, and the thermal stability had advantageously improved, especially at elevated temperatures. Compared to pure cotton, the T_max_ of sample CO/DOPO-HQ@Zr-MOF-10 was increased from 479 to 484.5 °C, and the R_max_ was decreased from 93.4 to 76.4 wt%/min. Meanwhile, the final char yield at 800 °C significantly increase from −1.21 to 4.85 wt%. Conclusively, depending on the fire performance of pure cotton and treated samples, DOPO-HQ@Zr-MOF composites were anticipated to work as a potential flame-retardant for cotton textiles. More importantly, the eco-friendly development of fire protection for cotton fabrics could substantially reduce fire hazards and extend evacuation time.

## Figures and Tables

**Table 1:** The treatment data of cotton fabrics by DOPO-HQ@Zr-MOF dispersions.

Sample	Dip-Dry (Cycles)	W_0_ (g)	W_1_ (g)	Add. wt (%)
CO/DOPO-HQ@Zr-MOF-1	1	5.68	5.84	2.82
CO/DOPO-HQ@Zr-MOF-10	10	5.82	6.52	12.03

**Table 2:** Summary of TGA data for cotton samples under an air atmosphere *.

	T_10%_ (°C)	Stage 1		Stage 2		Residue at 800 °C (wt%)
Sample		T_max_ (°C)	R_max_ (wt%/min)	T_max_ (°C)	R_max_ (wt%/min)	
Untreated cotton	318.5	341.6	37.6	479	93.4	−1.21
CO/DOPO-HQ@Zr-MOF-1	305.9	321.9	37.8	485	90	0.77
CO/DOPO-HQ@Zr-MOF-10	296.8	320.2	38.8	484.5	76.4	4.85

* The heating rate is fixed by 10 °C/min. T_10%_ is the initial decomposition temperature at which 10% of the sample weight is lost. T_max_ is the temperature of the maximum rate of weight loss. R_max_ is the weight loss rate at the maximal peak (T_max_).

## Data Availability

The data presented in this study are available on request from the corresponding author.
